# Resident and early-career family physicians’ focused practice choices in Canada: a qualitative study

**DOI:** 10.3399/BJGP.2021.0512

**Published:** 2022-03-08

**Authors:** Monisha Kabir, Ellen Randall, Goldis Mitra, M Ruth Lavergne, Ian Scott, David Snadden, Lori Jones, Laurie J Goldsmith, Emily G Marshall, Agnes Grudniewicz

**Affiliations:** University of Ottawa, Ottawa, ON, and research associate, Division of Palliative Care, Bruyère Research Institute, Ottawa, ON.; School of Population and Public Health, University of British Columbia, BC.; Department of Family Practice, Faculty of Medicine, University of British Columbia, BC.; Department of Family Medicine, Primary Care Research Unit, Dalhousie University, NS.; Department of Family Practice, Faculty of Medicine, University of British Columbia, BC; Director, Centre for Health Education Scholarship, Faculty of Medicine, University of British Columbia, Vancouver, BC.; Department of Family Practice, Faculty of Medicine, University of British Columbia, BC. Northern Medical Program, Prince George, BC.; Department of History, University of Ottawa, and Carleton University, Ottawa, ON.; GoldQual Consulting, and adjunct professor Faculty of Health Sciences, Simon Fraser University, BC.; Department of Family Medicine, Primary Care Research Unit, Dalhousie University, NS.; Telfer School of Management, University of Ottawa, Ottawa, ON.

**Keywords:** family practice, general practice, health policy, internship and residency, primary health care, qualitative research

## Abstract

**Background:**

Focused practice within family medicine may be increasing globally, but there is limited research on the factors contributing to decisions to focus practice.

**Aim:**

To examine the factors influencing resident and early-career family physician choices of focused practice across three Canadian provinces.

**Design and setting:**

A subset of qualitative interview data were analysed from a study across British Columbia, Ontario, and Nova Scotia, Canada.

**Method:**

Included in the analysis were a total of 22 resident family physicians and 38 early-career family physicians in their first 10 years of practice who intend to or currently practise in a focused area. Comparisons were made for participant types, provinces, and the degree of focused practice, while identifying themes related to factors influencing the pursuit of focused practice.

**Results:**

Three key themes were identified of factors contributing to choices of focused practice: self-preservation within the current structure of the healthcare system; support from colleagues; and training experiences in medical school and/or residency. Minor themes included: alignment of practice with skills, personal values, or ability to derive professional satisfaction; personal lived experiences; and having many attractive opportunities for focused practice.

**Conclusion:**

Both groups of participants unanimously viewed focused practice as a way to circumvent the burnout or exhaustion they associated with comprehensive practice in the current structure of the healthcare system. This finding, in addition to other influential factors, was consistent across the three provinces. More research is needed to understand the implications of resident and early-career family physician choices of focused practice within the physician workforce.

## INTRODUCTION

In recent decades, there has been a global decline in offering a comprehensive scope of practice in family medicine,^1^^–^6 and a concurrent trend towards focused practice,^7^^–^13 wherein one or more specific clinical areas form a major part-time or full-time component of practice.^14^ Previous research suggests that this trend is in part due to perceptions that focused practice offers a desirable intellectual challenge^8^ and better remuneration.^15^ Characteristics such as the region of intended practice^16^ and being a male non-parent^17^ have also been identified as potential influences for focused practice choices. Other studies suggest that the exodus from comprehensive family medicine practice globally can be attributed to both the breadth and the overwhelming nature of its scope,^7^^,^^12^^,^^18^ and undesirable post-training working environments.^3^ However, there are few studies that have provided an in-depth examination of the diverse factors influencing the pursuit of focused practice in family medicine. To the authors’ knowledge, there is only one study to date that has broadly examined factors contributing to family physician (FP) scope of practice choices.^19^ That study identified personal, workplace, environment, and population elements shaping local FP-focused practice decisions in one state in the US. This current study builds upon the results of the previous study from the Canadian perspective, using interviews with both resident and independently practising FPs. There is also discussion of the international relevance of the findings.

The objective of this study was to examine the factors contributing to choices of focused practice in three Canadian provinces. In Canada, medical school is graduate entry, lasts 3 to 4 years, and for family practitioners is followed by 2 years as a resident FP before certification as an independent family practitioner. Findings are presented from both resident FPs and independent family practitioners in their first decade of practice, henceforth ‘early-career FPs’, to address the research question.

**Table:** How this fits in

Internationally, family physicians are increasingly turning towards focused practice options at the expense of offering comprehensive family medicine. Other studies have identified possible factors contributing to focused practice choices. However, few studies have examined the range of factors influencing focused practice choices in depth. This study explores these influential factors in three Canadian provinces using the perspectives of resident and early-career family physicians, and highlights the need for policy changes and further research in this area on an international scale.

## METHOD

### Study design and population

The study reports on a subset of the qualitative interview data collected through a larger mixed-methods study examining factors contributing to practice intentions and choices among resident and early-career FPs across British Columbia, Ontario, and Nova Scotia, Canada. The details of this study are available in the published protocol.^20^ This paper was an a priori planned component of this larger study.

Resident and early-career FPs were recruited for the qualitative arm of this larger mixed-methods study through family medicine residency programme email listservs in all three provinces, social media (Twitter and Facebook), and one provincial medical association (Doctors of Nova Scotia).

Interested participants completed an online screening questionnaire that captured demographic information and practice characteristics (Supplementary Box S1) and was developed and pilot-tested by the research team for this study. Purposeful sampling was used to maximise variation across self-identified sex, marital status, dependants, training location, years of training, years in practice, scope of practice, and practice models in each province. Selected individuals were then invited to participate in a 60-min interview. Participants were provided with study information and an honorarium.

There were interviews with 31 of 32 resident FPs and 63 of 69 early-career FPs who had been invited to participate in the study. Reasons for non-participation included scheduling conflicts (*n* = 2), no response (*n* = 4), or withdrawal with no reason provided (*n* = 1). The study received ethical approval in all three provinces.

### Context for this paper

The sample used in this paper consisted of 22 resident FPs and 38 early-career FPs who: 1) self-identified in the screening survey as intending to practise or currently practising within a focused area; and/or 2) described focused practice elements in their overall practice during the interview. Resident and early-career FPs who self-identified as intending to offer (resident FPs) or currently offering (early career FPs) comprehensive family medicine services in addition to their focused practices were also included in the analysis. Resident and early-career FPs were considered to have a focused practice, in whole or in part, if a component of their intended or current practice was narrowed or specialised in scope (for example, addictions medicine, emergency medicine) and they did not intend to or currently deliver any routine comprehensive family medicine care to patients in that part of their practice. This conception of focused practice is consistent with existing definitions describing focused practice as one or more specific clinical areas forming a major part-time or full-time component of an FP’s overall practice.^14^ This definition was further operationalised, based on the data from resident and early-career FPs, to add that routine comprehensive family medicine care was not delivered to patients in the participants’ focused practices. This part of the definition used for focused practice was data driven. Resident and early-career FPs were considered to be engaging in comprehensive family medicine practice when they described providing clinic-based, first-contact, longitudinal, and coordinated services to a defined group of patients to address the majority of their healthcare needs.^21^^,^^22^ This study’s definition of comprehensive family medicine practice included providing comprehensive care for a particular population (for example, refugees) or comprehensive care omitting obstetrics/prenatal care.

### Data collection

One research analyst per province conducted one-on-one, semi-structured, in-depth interviews. Each research analyst was trained in qualitative interviewing. Telephone interviews were conducted using a semi-structured interview guide specific to each subgroup (Supplementary Box S2). Interviews were audio-recorded and transcribed verbatim. Research analysts recorded their reflections and interview summaries after each interview. Participant recruitment occurred iteratively until no new themes were identified in interviews.

### Data analysis

The generic qualitative inquiry^23^ approach was used in this study to understand a pragmatic issue: the factors contributing to resident and early-career FP decisions to pursue focused practice. This method of inquiry does not require researchers to ascribe to a specific qualitative tradition when attempting to address a practical, clear-cut question.^23^

Iterative, inductive thematic analysis was used.^24^ For the qualitative arm of the larger mixed-methods study, three research analysts with experience in qualitative analysis generated initial resident and early-career FP codebooks through inductive coding of one resident and one early-career FP interview.^25^ Codebooks were then refined through application to a subset of transcripts with guidance from the senior author. The research analysts used the final codebooks to code transcripts from their respective provinces in NVivo 12. Codebooks were iteratively amended to incorporate emerging codes and ensure consistency between the resident and early-career FP codebooks. The research analysts also coded one interview from another province to ensure reliability.

Data analysis for this article involved two researchers, reviewing interview excerpts coded as influential factors for practice choices and aggregating them into potential overarching themes, based on the identification of patterns between and across the transcripts. Themes were presented to FP members of the research team throughout the process for feedback on the findings. Comparative analysis^26^ was conducted to compare thematic patterns identified from the early-career FP transcripts with the resident FP dataset. If new themes were identified from the resident FP transcripts, an iterative approach was used to find corresponding themes in the early-career FP transcripts. Themes across provinces were also compared.

The trustworthiness of the data analysis was ensured using multiple strategies, including:

- triangulating across a large sample size of participants with diverse experiences from three provinces and two types of participant groups;^27^^,^^28^
- conducting data collection and analysis in iterative ways using multiple analysts;^29^ and
- presenting to FP members of the research team, who were not involved in the analysis process for this paper, throughout analysis to confirm data interpretation.^28^

## RESULTS

The 22 resident and 38 early-career FPs reported a variety of intended or current clinical areas of practice, with most participants combining focused area(s) and/or some form of comprehensive practice and focused practice. Demographic and practice characteristics are presented in Tables 1 and 2 to reflect the range of resident and early-career FPs included in the analysis.

**Table 1.** Planned practice characteristics of resident FP participants choosing to focus their practice (*N* = 22)

**Characteristic**	***n* (%)**
**Province**	
British Columbia	7 (32)
Ontario	6 (27)
Nova Scotia	9 (41)

**Planned area of clinical practice^a^**	
**Focused practice**	22 (100)
Emergency medicine	13 (59)
Hospitalist medicine^b^	9 (41)
Addiction medicine	7 (32)
Sexual and reproductive health	4 (18)
Obstetrics and maternity care	3 (14)
Medical aesthetics, dermatology	3 (14)
Geriatrics	2 (9)
Mental health	2 (9)
Palliative care	2 (9)
Sports medicine	2 (9)
Oncology	1 (5)
**Comprehensive family medicine practice**	21 (95)
Comprehensive family medicine practice	13 (59)
Comprehensive family medicine practice with no obstetrics/prenatal care	5 (23)
Comprehensive family medicine practice for a particular population	3 (14)

a *Planned areas of clinical practice are not mutually exclusive as most participants’ intended practices incorporated a combination of these elements.*

b *Refers to FPs exclusively delivering care within a hospital setting.*

**Table 2.** Practice characteristics of early-career FP participants with focused practices (*N* = 38)

**Characteristic**	***n* (%)**
**Province**	
British Columbia	14 (37)
Ontario	13 (34)
Nova Scotia	11 (29)

**Area of clinical practice^a^**	
**Focused practice**	38 (100)
Hospitalist medicine^b^	14 (37)
Emergency medicine	12 (32)
Sexual and reproductive health	9 (24)
Obstetrics and maternity care	5 (13)
Surgical/procedural medicine	4 (11)
Addiction medicine	3 (8)
Medical assistance in dying (MAiD)	3 (8)
Travel medicine	2 (5)
Urgent care	2 (5)
Student health, youth mental health	2 (5)
Consultation for provincial workers’ safety board	1 (3)
Medical aesthetics, dermatology	1 (3)
Palliative care	1 (3)
Sports medicine	1 (3)
**Comprehensive family medicine practice**	30 (79)
Comprehensive family medicine practice	22 (58)
Comprehensive family medicine practice with no obstetrics/prenatal care	4 (11)
Comprehensive family medicine practice for a particular population	2 (5)
**Non-clinical**	2 (2)
Academic/administrative	2 (2)

a *Areas of clinical practice are not mutually exclusive as most participants’ practices incorporated a combination of these elements.*

b *Refers to FPs exclusively delivering care within a hospital setting.*

Among the resident FPs who intended to focus their practices, 21 (95%) anticipated practising some form of comprehensive family medicine with focused practice. One resident FP envisioned solely working in focused practice. Among early-career FPs, 21 (55%) devoted more than half or all their time to a focused area and 30 (79%) offered some form of comprehensive family medicine. The most commonly reported areas of focused practice in both groups were emergency and hospitalist medicine (refers to physicians exclusively delivering care within a hospital setting in Canada).^30^ Focused practice choices occurred on a continuum, ranging from the provision of all services under the umbrella of a defined area (for example, dermatology) to a specific procedure within a particular area (for example, only Botox injections).

### Key factors contributing to intentions or choices of focused practice

Three key and three minor themes of influential factors that helped explain participants’ decisions to pursue focused practice were identified. Key themes were prominent across both resident and early-career FP datasets, while minor themes were less salient in the data.

#### Self-preservation within the current structure of the healthcare system

Both participant groups described issues within the healthcare system that influenced their choices of focused practice, specifically with regards to remuneration and workload. Certain physician remuneration models, such as fee-for-service, deterred participants from practising comprehensive family medicine. Fee-for-service was seen as inadequate compensation for the long hours, workload, and overhead costs associated with longitudinal care for increasingly complex patients. One resident FP elaborated:

*‘It’s a bit of a crisis. I feel like a lot of physicians are burnt out … And, you know, documentation also takes up time with forms and everything. And I feel like … that’s not really being considered. And when it comes to the fee-for-service model, that’s why I don’t think it would work for me just because patients are a bit more complex than they used to be … Like I don’t think you should be rushing through your patients or just having single issue appointments … So I think when they’re* [the government] *making their policies and doing the compensation and payment plans, I’d like to see them sort of consider that …’*

(R14, Nova Scotia)

In contrast, focused practice was seen as more attractive and sustainable because of better compensation and fewer administrative costs. The early-career FPs in this study described policies governing primary care delivery in all three provinces as contributing to heavy workloads and concerns about burnout. Similarly, resident FPs relayed observations of FP mentors being overworked, inadequately remunerated, and having difficulty securing time off in comprehensive family medicine practice. In contrast, both participant groups felt that focused practice offered better remuneration and flexibility to choose hours worked, allowing more time for family commitments, hobbies, or parental leave. A resident FP highlighted the advantages to focused practice:

*‘Overhead is not something that you have when you work as a hospitalist … So you definitely make more money than you would in a clinic setting in a big city … the main thing about hospitalist work is that again it doesn’t come attached with you taking care of an office, of a staff … And then once you’re done your week of work, you don’t have the patients to follow after that.’*

(R2, British Columbia)

Resident and early-career FPs also saw parental leave as incompatible with comprehensive family medicine practice. An early-career FP summarised the obstacles presented by parental leave:

*‘If I were to take something like maternity leave … you don’t want to have 2000 patients* [in a comprehensive family medicine practice] *and then have to go off for a year … or however long you’re on maternity leave. And so that would make me … kind of think like do I actually want to take on patients? Or is that something I’d want to do after, you know, in ten years when I feel like I’ve had a family and I’m back to working full time? Or is it something that I just don’t want to do because as soon as you have a roster of patients, it makes it very difficult to leave or to move or to change your mind as much … Like I would like to have more flexibility in terms of taking time off … And finding locums is a little challenging …’*

(FP26, British Columbia)

Other challenges described by both participant groups in reference to parental leave in comprehensive family medicine practice included perceived resentment from patients for time off and interruptions in patient continuity of care.

Further, early-career FPs with focused practices described feeling pressured during their training to work in what they considered an antiquated FP role. They shared that instructors put emphasis on a traditional paradigm of comprehensive family medicine practice that involved working around the clock to serve patients and that this was the best way to practise. An early-career FP explained this further:

*‘There’s such a huge generational gap in medicine. And you know, the generation that by and large is training us just doesn’t see another way to be … But they truly think … that people doing focused practices are providing inferior care … This generation of doctors, we’re not lazy and we don’t not care about patients. We’re just not willing to ruin the rest of our lives for the career. And it’s self-preservation. We care about people too. We* [are] *also not willing to lay down our lives for the system.’*

(FP4, British Columbia)

Early-career FP participants perceived these traditional comprehensive FP roles as unachievable for current and future levels of patient complexity and need, and detrimental to their wellbeing and families. Resident and early-career FPs alike expressed an unwillingness to sacrifice work–life balance, believing that policy reform was necessary for them to consider a broader scope of practice. Both participant groups were unanimously dissatisfied with provincial government policies and considered their governments to be unresponsive to their needs and undervaluing FPs.

#### Access to a support system

Resident and early-career FPs felt focused practice offered greater access to a support system compared with comprehensive family medicine practice. Both participant groups viewed call groups and team-based care environments within focused practice areas (for example, hospitalist medicine) as support systems that improved quality of care, facilitated knowledge sharing, and decreased isolation. One resident FP elaborated:

*‘Working in hospitals, I think it’s a huge advantage over working in clinics in terms of multidisciplinary work. You know, in hospital, you basically have all the different specialties … Which is awesome and I kind of like that teamwork. Whereas clinics, when you work in a family practice office, unless it’s a big clinic and they have the multidisciplinary team, I find most clinics will have maybe one nurse or two … So yes, of course, I really like working with other specialists. I think it makes your life much easier and it helps us to provide better care. And it’s one of the reasons why hospitalist, for example, is more attractive to me.’*

(R2, British Columbia)

These support systems facilitated self-preservation in the current health care system. Early-career FPs also described their peers as role models who demonstrated the feasibility of incorporating focused areas into their overall practices.

#### Training experiences

Both participant groups reported training experiences that increased their comfort with focused areas of practice and created recognition that the workload in comprehensive family medicine was not an ideal match for their desired lifestyle. An early-career FP illustrated this:

*‘It felt most of the doctors that I followed* [in medical school] *, you know, would see thirty to forty patients a day. They were mostly older white men … the physician I followed would see up to fifty patients a day … And it was exhausting. And I don’t think I saw myself in a model like that. And so even though I chose family, I think in my mind I knew I wasn’t going to practice in that manner.*’

(FP19, Ontario)

This belief was reinforced by resident and early-career FP perceptions that their mentors were exhausted in comprehensive family medicine practice environments.

### Minor themes for influential factors for focused practice

Resident and early-career FPs described feeling attracted to a particular focused area (for example, hospitalist medicine) because it aligned with their skills/values or helped them maintain specific competencies (for example, high-acuity skills). Other reasons for choosing focused practice included increasing variety in their work or being intellectually stimulated. Both participant groups indicated that a focused practice brought with it a sense of professional satisfaction by filling a perceived gap in care. One early-career FP highlighted this:

*'* [What is most important to me in my career is] *that I feel good about the work that I’m doing. That I feel like I’m contributing to my community in a way that helps people … Maybe just what was needed in our community … MAiD* [medical assistance in dying] *was something that was under-serviced. And the woman that was only doing it at the time was quite stressed out. And when she approached me, it just made sense to do it.’*

(FP22, British Columbia)

Resident FPs described being attracted to focusing their practice because of available opportunities, community needs, and limited specialist availability. One resident FP elaborated:

*‘For more specific things like dermatology, I know there’s always sort of a very long wait list to see a dermatologist. So I think, at least from what I’ve seen, anyone who’s kind of had a focused interest in that, there’s no shortage of people or patients coming to see you.’*

(R8, Nova Scotia)

Similarly, early-career FPs reported being inclined to incorporate focused areas into their practices because of a multitude of job opportunities in focused practice.

Both participant groups also described personal lived experiences that sparked their interest in particular focused practice areas. For example, experiences with family members, friends, or community members with mental health struggles or addictions contributed to interests in focused practices in mental health and addictions medicine, as one resident FP described:

*‘As a teenager, late teenager, a lot of my friends got quite heavily into drugs and then selling and doing drugs … And I think that’s a big reason why I’m drawn to addictions as well — watching them go through that and be arrested and go to jail. These people I’ve known for seven, eight, nine years. And their life took a huge nosedive that they’re only now recovering from, is a big reason why I’m drawn to addictions.’*

(R16, Nova Scotia)

Similarly, prior volunteer experiences also shaped resident and early-career FP choices of focused practice.

### Comparison between provinces

These results were comparable across the provinces studied. Resident and early-career FPs in British Columbia and Nova Scotia described similar concerns about inadequate compensation for the workload and responsibility involved in comprehensive family medicine practice. Specifically, early-career FPs in these provinces desired fee-for-service fee schedules that aligned with other provinces or alternative payment models. In Ontario, resident and early-career FPs expressed dissatisfaction with the province’s numerous payment models, describing loss of control over earnings in comprehensive family medicine practice, uncertainty, and distrust due to a fluctuating policy landscape (for example, fee cuts, role restrictions).

## DISCUSSION

### Summary

The interview participants in this study found focused practice attractive for numerous reasons, including: more manageable workloads, better remuneration, and improved work–life balance; familiarity from prior exposure during training; and the presence of a supportive team environment. Less common reasons for opting for focused practice included alignment with participants’ skills, values, or an ability to feel professional satisfaction; personal lived experience; and having a multitude of opportunities to practise. Resident and early-career FPs described focused practice as a way to circumvent burnout or exhaustion, which they considered to be an untenable component of comprehensive family medicine practice in the current healthcare system. Discontent with provincial policies and the lack of government responsiveness to their concerns was apparent across all provinces and practice types.

### Strengths and limitations

Data collection for this study occurred prior to the onset of COVID-19 and therefore does not necessarily reflect the current environment. This study only includes individuals who responded to requests to participate, which may not reflect all types of resident and early-career FPs. Participants were not asked specific questions about sex/gender, geography, and training location, focusing instead on open-ended questions. A richer description may have developed by probing these additional areas. Strengths of this study are that it corroborates influential factors for focused practice previously identified in the literature, and describes additional elements that, to the authors’ knowledge, have not been described before. Moreover, similar factors were found to contribute to choices of focused practice for both resident and early-career FPs, demonstrating that these factors are consistent prior to and during independent practice.

### Comparison with existing literature

Previous work has identified newly graduating resident FPs as more likely to intend to provide a broad scope of practice compared with FPs in current practice.^3^^,^^5^^,^^31^^,^^32^ Though this current study was not designed for statistical comparisons, it also found that resident FPs were more likely to report intending to practise comprehensive family medicine than early-career FPs. The post-training working environment and lack of support for providing a broad scope of services have been suggested as possible driving forces for this finding,^3^^,^^19^^,^^32^ contributing to resident FPs being deterred from offering comprehensive family medicine once they begin practising. Other studies from Belgium, France, the UK, and US have described comprehensive family medicine practice as too broad, overwhelming, involving a high degree of responsibility, allowing for minimal work–life balance,^7^^,^^8^ and providing insufficient financial incentives.^9^ Factors reported to support the choice of focused practice include superior financial incentives,^15^ opportunities for intellectual stimulation,^7^^,^^8^ greater work-life balance,^19^ reduced stress from a lower workload,^7^ community needs,^19^ and prior training exposures.^19^^,^^33^ This current study confirms these findings. FPs in Canada and the US have reported feeling unprepared to deliver comprehensive care.^12^^,^^32^^,^^34^ This was not found in this study. Instead, system-level barriers were found, linked to government policy, influencing focused practice choices.

The shift to focused practice has been occurring on an international scale.^7^^–^13 Between 2015 and 2019 alone, FPs providing comprehensive family medicine across Canada and 10 other developed countries in the Commonwealth Fund (Australia, France, Germany, the Netherlands, New Zealand, Norway, Sweden, Switzerland, the UK, and the US) have increasingly described their work as stressful.^35^ This current study supports the link between resident and early-career FP choices of focused practice due to their perceptions of comprehensive family medicine practice being overwhelming. This finding suggests that some of the influential factors for focused practice choices identified in this study may be implicated in the changes in FP practice patterns on an international scale.

### Implications for research and practice

There is contention about the benefits and harms of increasing focused practice within family medicine.^36^ The impact of rising numbers of FPs practising in focused areas on the supply of FPs providing comprehensive care is still unknown globally and requires more research. Future studies on focused practice may wish to build on this study's findings to confirm or further explore the factors identified here, as well as other factors that may appear in response to contextual changes in local health policy environments internationally. Further work is also needed to identify reforms, such as additional funding support and clear policies,^18^ which may encourage FPs to offer a comprehensive scope of family medicine while supporting their personal and professional wellbeing.

Given the importance of comprehensive family medicine, coupled with the global trend towards focused practice, it is critical that we further understand the impact on healthcare services in Canada and internationally to ensure the sustainability and growth of a robust primary care system.
